# Influences of Errors in Modular-Assembled Antenna on Radiation Characteristics

**DOI:** 10.3390/s25144244

**Published:** 2025-07-08

**Authors:** Huanxiao Li, Shengnan Lyu, Xiaofei Ma, Yu Shi, Zexing Yu, Xiuji Chen, Xiaotao Zhou

**Affiliations:** 1 School of Mechanical Engineering and Automation, Beihang University, Beijing 100191, China; lihuanxiao2015@126.com (H.L.); shengnan_lyu@buaa.end.cn (S.L.); by2407131@buaa.edu.cn (Y.S.); 2 Xi’an Institute of Space Ratio Technology, Xi’an 710100, China; yuzexing@mail.nwpu.edu.cn (Z.Y.); xiuji1934@163.com (X.C.); zxtemc_2015@163.com (X.Z.)

**Keywords:** influence, modular assembled, errors analysis, radiation characteristics

## Abstract

Modular-assembled antennas represent an effective solution for the challenge of building super-large antennas in orbit. To investigate the impact of errors in modular-assembled antennas on their radiation characteristics, this study proposes definitions for these errors and presents methods for addressing them during the engineering design phase. The sources of errors in the modular antenna units are identified, and formulas for the error contributions of each module are derived. Based on this error analysis, a relationship between the errors of individual modules and the overall assembled antenna is established, along with an analytical expression for the antenna’s error. The influence of various error terms on the radiation characteristics of the assembled antenna is then examined. Simulations of the antenna’s radiation performance have been conducted, and the results demonstrate that changes in the antenna’s error patterns correlate with variations in its radiation characteristics. These findings provide valuable insights for guiding the engineering design of modular-assembled antennas.

## 1. Introduction

As a crucial component of modern communication technology, the mesh deployable antenna offers several advantages, including a large aperture, foldability, and lightweight design. These characteristics make it widely applicable in fields such as detection, communication, and remote sensing [1,2]. Given the diverse requirements of space missions, various types of mesh deployable antennas have been developed. Common designs include ring antennas, umbrella antennas, frame antennas, and ETS-VIII frame antennas [3,4]. Most existing mesh deployable antennas are designed for single deployment, allowing them to be compactly stowed in a rocket’s payload bay during launch and later deployed into their operational configuration once in orbit. However, with the rapid advancement of space technology, these conventional designs can no longer fully meet the demands of certain space missions.

Larger apertures and higher surface accuracy are key development trends for mesh deployable antennas. To meet the application demands for large-aperture antennas, an advanced space technology—on-orbit assembly of antennas—has been proposed. This technology involves the in-space assembly and integration of individual antenna modules or components, overcoming the limitations imposed by launching large antennas from the ground. Through modular design and on-orbit assembly, this approach enables the realization of larger-scale antenna systems.

On-orbit antenna assembly technology encompasses several key aspects, including module unit design, docking mechanism design, robotic assembly techniques, and on-orbit measurement and adjustment technologies [5,6,7,8,9,10,11,12]. In the domain of modular-assembled antenna design, Li et al. [7] proposed a modular element design method tailored to common spatial surface structures. Dong et al. [9] introduced a design method for assembled antenna nodes that accounts for structural gaps and incorporates actuators to correct on-orbit errors. Meguro et al. [12,13] developed and tested a typical ETS-VIII modular antenna in orbit. Guo et al. [14] conducted a dynamic study on modular truss structures and developed a prototype for comparative verification. Song et al. [15,16,17] proposed a novel truss module for a space-assembled telescope (iSAT), simplifying the assembly process and reducing manufacturing costs. Ma et al. [18] designed a geometrically extensible hexagonal prism module for a deployable parabolic cylinder antenna, considering both topological and structural aspects. Tian et al. [19] explored a deployable antenna structure composed of hexagonal and pentagonal prisms, analyzing its kinematic characteristics. Zhang et al. [20] proposed a design for a parabolic cylindrical deployable antenna based on cable-rib tension structures, which was verified through a physical prototype. Deng et al. [21] proposes a modular assembled mesh antenna constituted by several hexagonal platform units. Dong et al. [22] proposed a method for dynamic modeling and attitude control for the on-orbit assembly process of modular antennas. Shi et al. [23] designed a brand-new assembly interface for large space structures and analyzed its performance in detail according to the requirements of on-orbit assembly.

Further advancements have been made in mesh geometry and modular design. Li et al. [24] proposed a mesh geometry design method that considers electrical properties to enhance antenna gain performance under given surface error constraints. Sun et al. [25] analyzed the random errors in the manufacturing process and their impact mechanism on the antenna surface accuracy. Yuan et al. [26] proposed an improved pretension design method for mesh reflector antennas based on the equilibrium matrix method (EMM), aiming to address the limitation of traditional methods in accurately considering truss and hinge deformations, which adversely affect the surface accuracy of the antenna. Wu et al. [27] proposed a novel design method based on a rib-mesh structure to address the challenge of ensuring surface accuracy in deployable paraboloidal antenna reflectors. Yu et al. [28] proposed an active optimization adjustment method to ensure the surface accuracy of spacecraft antennas under varying thermal loads. Li et al. [29] developed an interval force density method for mesh antennas to analyze the influence of cable uncertainty on surface accuracy. Chen et al. [30] utilized area coordinates to derive the mathematical relationship between the axial principal error of reticulated antennas and geometric surface parameters. Li et al. [31] proposed a design methodology for shaped mesh antennas, optimizing the pretension distribution for enhanced performance. Tang et al. [32] investigated an analytical approach to mitigate the inverse pillow effect in mesh antennas, examining its impact on surface accuracy and electrical performance.

Among these studies, extensive research has been conducted on mesh antennas, primarily focusing on structural mechanism design, mesh surface modeling, dynamic analysis, and error estimation. Investigations into modular antenna technology have largely concentrated on hexagonal prism-based configurations, truss element design, and kinematic and dynamic analyses. However, research on modular-assembled antennas remains limited, with most studies emphasizing modular unit design and kinematics analysis. Despite the critical role of modular-assembled antennas in future engineering applications, few studies have explored their comprehensive design and error evaluation.

In this paper, we propose a systematic error definition for modular-assembled antennas at the engineering design level. Through a formula-based analysis of various error sources, we derive the overall error for a modular-assembled antenna system. The effects of different error components are examined, and a relationship between gain performance and error variations is established. This study provides valuable guidance for the engineering application of modular-assembled antennas, contributing to their future development and deployment.

## 2. Methods

### 2.1. Antenna Structure Components

The key feature of the modular assembly antenna is its ability to be deployed in orbit, meeting the future demand for 100 m or even kilometer-scale aperture antennas in space. The main design concept involves dividing the target antenna structure into modules of a specific scale and designing each module individually.

During launch, each module is compactly stowed within the rocket’s payload compartment. Once in the target orbit, the module expands into an independent structural unit. These modules are then interconnected via a docking mechanism, allowing for the gradual expansion of the overall antenna structure.

**Figure 1:**
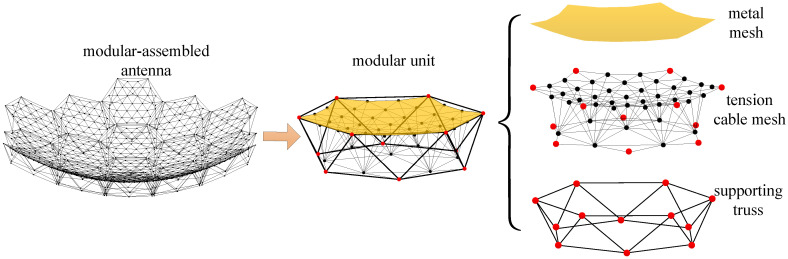
Composition of modular-assembled antenna.

The modular assembly antenna consists of multiple modular units, with each unit comprising three main components: a metal mesh, a tension cable-net, and a support truss, as illustrated in Figure 1.

The metal mesh, commonly used in mesh deployable antennas for reflecting electromagnetic waves, is typically woven from gold-plated molybdenum wire. The tension cable-net is a flexible structure that conforms to the reflector surface of the mesh antenna, enabling the approximation of different reflective surfaces through cable tensioning. The supporting truss serves as the primary structural component of the modular unit, facilitating both deployment and folding.

The modular assembly antenna is composed of multiple modular units, each relying on a metal mesh to achieve electromagnetic wave reflection. The design methodology for a single modular unit has been explored in previous studies [8], where the truss nodes are positioned on a standard parabolic surface. However, the design and assembly errors of the truss structure are not considered.

**Figure 2:**
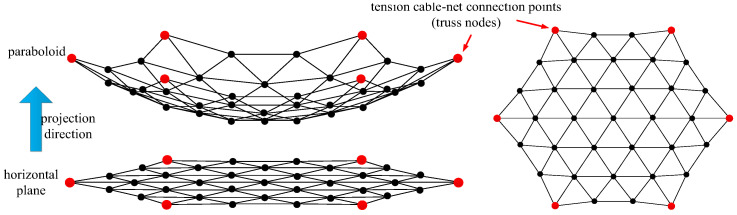
Schematic diagram of the primary reflector surface.

In this paper, the design of the cable-net system for modular units focuses solely on the front net of the tension cable-net. The metal mesh is sewn onto this front net, and the primary reflector surface is generated using the projection method [30], as illustrated in Figure 2.

In Figure 2, the primary reflector surface is segmented on the horizontal plane and then projected onto the standard parabolic surface to determine the required nodes and connections. Since the modular units must be capable of folding and deploying, the tension cable-net is restricted to connections at only six nodes of the supporting truss.

**Figure 3:**
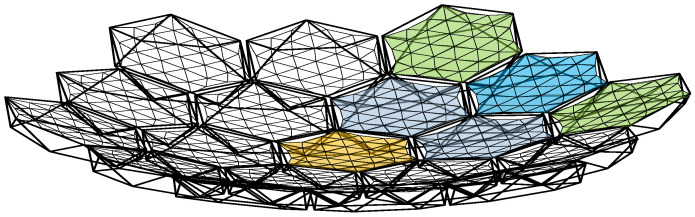
Schematic diagram of a modular-assembled antenna.

**Figure 4:**
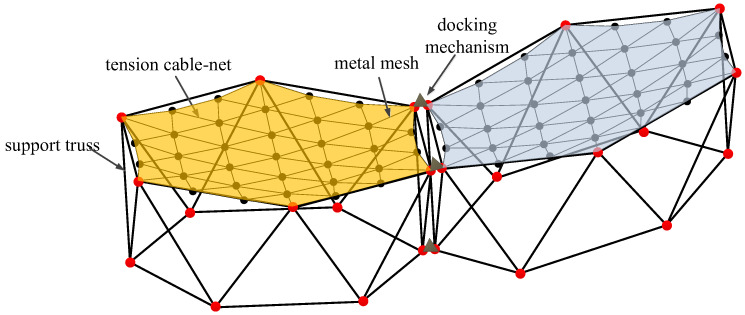
Connection of two modules.

Additionally, to maintain force balance in the primary reflector, the outer edge of the metal reflector must exhibit a certain degree of sag [33]. When assembling the modules, a docking mechanism is employed to interconnect them. The fully assembled antenna is illustrated in Figure 3, while Figure 4 depicts the connection between two modules.

### 2.2. Sources of Errors

As a transceiver of electromagnetic waves, the accuracy of the reflector surface and the aperture area significantly influence the antenna’s electrical performance. The accuracy of the reflector surface is mainly affected by errors introduced when approximating a standard parabolic surface using triangular facets. Likewise, the projected area of the reflector onto the aperture plane is a key factor impacting the antenna’s gain performance.

Existing error models for unassembled mesh antennas are not fully applicable to modular-assembled antennas. Therefore, in this study, we define a new error model specific to modular-assembled antennas. Considering the area loss of the metal reflector surface and the physical gaps between modular units, we analyze errors that arise during the design phase. Three types of errors are identified and described as follows:

#### 2.2.1. Module Surface Error

The surface error of a mesh antenna primarily results from the deviation between the small triangular facets and the ideal parabolic surface after tensioning. This error is quantified using the root mean square (RMS) of surface deviation, as expressed in Equation (1).

(1) $$\delta =\sqrt{\frac{1}{n}\sum_{i=1}^{n}{\left(z_{i}-z\right)}^{2}}$$

where $z_{i}$ (mm) represents the deviation of the centroid of the *i*-th triangle from the theoretical surface along the antenna’s focal axis, and z (mm) is the average of all $z_{i}$ values.

**Figure 5:**
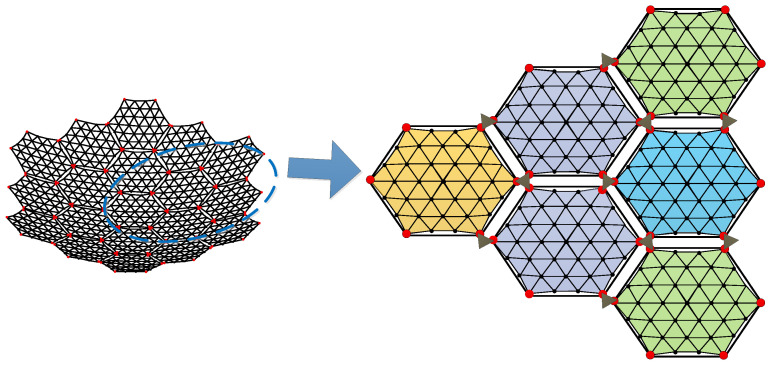
Module surface error.

The definition of surface error for a single module in the assembled antenna is consistent with that of a conventional mesh antenna. This error arises from the approximation of the parabolic surface using triangular facets formed by the tensioned cable-net within the modular unit. The accuracy of this approximation is directly influenced by the spacing of the mesh division—smaller grid spacing results in higher surface accuracy. Since the antenna is assembled using different modular units positioned at varying locations, the surface errors of individual modules are not identical, as illustrated in Figure 5.

Let $\delta_{m}$ represent the surface error of the *m*-th modular unit. In Figure 4, each set of six red nodes indicates the connection points between the tension cable-net and the supporting truss of a module. The surface accuracy of the module is expressed in Equation (2).

(2) $$\delta_{m}=\sqrt{\frac{1}{n}\sum_{i=1}^{n}{\left(z_{i}-z\right)}^{2}}$$

#### 2.2.2. Metal Mesh Area Error

**Figure 6:**
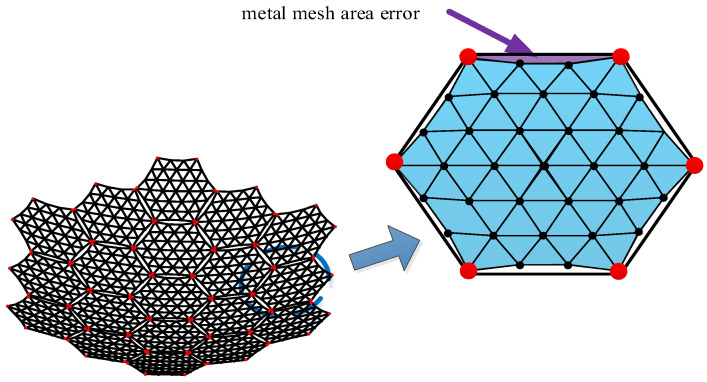
Metal mesh area error.

Metal mesh area errors are also present in existing single-deployed mesh antennas. Typically, this issue is mitigated using a compensation method; however, the compensated area does not effectively reflect electromagnetic waves. In a modular-assembled antenna, each unit contributes to the overall metal mesh area error. As the number of units increases, the accumulation of these errors becomes a significant factor affecting the overall antenna performance, as illustrated in Figure 6.

**Figure 7:**
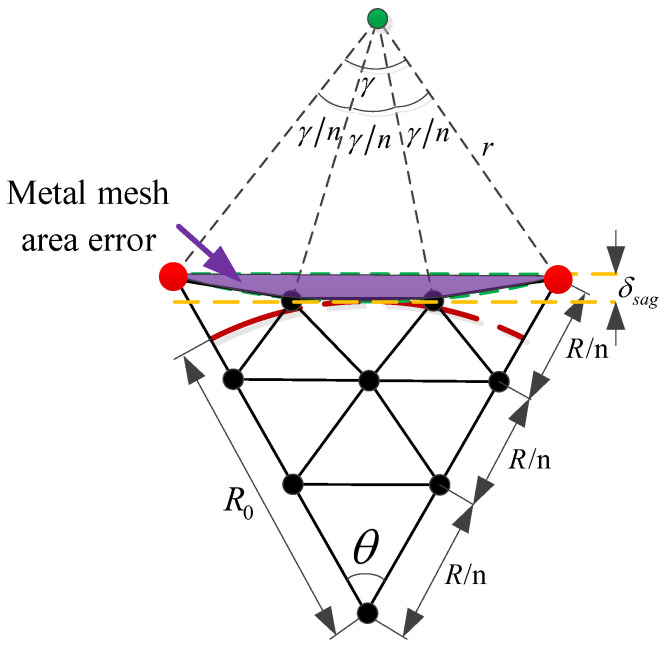
Calculation of the metal mesh area error.

The definition of metal mesh area error is illustrated in Figure 6. Since the metal mesh is sewn onto the tension cable-net, a certain sag-to-span ratio is required at the boundary to ensure uniform tension distribution across the cable-net. As a result, the metal mesh cannot fully cover the entire modular unit, leading to gaps that cause electromagnetic wave leakage. This uncovered area constitutes the metal mesh area error. Based on the relationship between a reflector antenna’s gain and its aperture area, the presence of metal mesh area error leads to a reduction in antenna gain. Figure 7 illustrates the calculation of this error.

As shown in Figure 7, 1/6 of the modular unit is taken for illustration. Once the size of the modular unit and the mesh surface division are determined, and given the sag-to-span ratio of the module [26], the metal mesh area error for a single module can be calculated as follows:

(3) $$S_{iJS}=3r^{2}(\gamma -\sin\left(\gamma \right))$$

where $S_{iJS}$ is the metal mesh area error of the *i*-th module; R is the projected side length of a single module; r is the radius of the circle required for the sag-to-span ratio, $r=(\delta_{sag}^{2}+R^{2}{sin}^{2}(\theta /2))/(2\delta_{sag})$; γ is the central angle of the circle, $\gamma =2arccos\;(r-\delta_{sag})/r$; $\delta_{sag}$ is the sag, usually given in the form of sag-to-span ratio $\rho =\delta_{sag}/(2R_{0}tan(\theta /2))$; $R_{0}$ is the effective area radius of the module; θ is the interior angle of the module, θ=π/3.

#### 2.2.3. Assembly Gap Error

**Figure 8:**
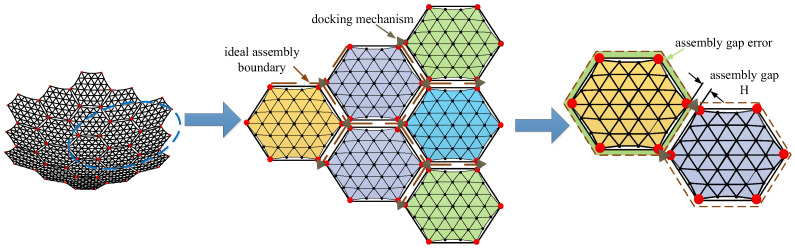
Assembly gap error.

Assembly gap error is a unique source of error in modular-assembled antennas. Since multiple modules are connected using docking mechanisms, the physical structure of these mechanisms inevitably results in slight misalignments between modules, creating assembly gaps. The area lost due to these gaps on the projected aperture of the reflector causes electromagnetic wave leakage, which in turn reduces the antenna’s gain performance. The assembly gap error is illustrated in Figure 8.

**Figure 9:**
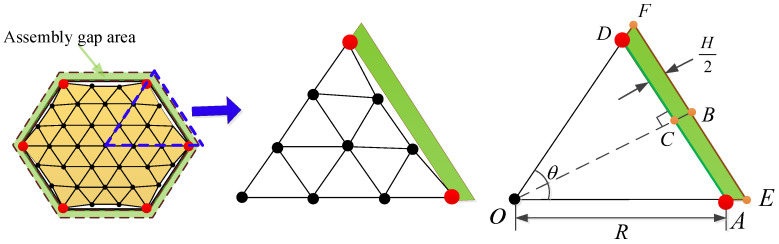
Calculation of assembly gap error.

In Figure 8, multiple modules are assembled together. Ideally, the supporting trusses of the modules would connect seamlessly, forming a perfect assembly boundary. However, due to the physical dimensions of the docking mechanism, an assembly gap is introduced between each pair of modules after docking. The size of this gap depends on the dimensions of the docking mechanism. When projected onto the aperture surface of the antenna, these gaps create discontinuities in the reflector surface. The resulting missing area reduces the reflective surface available for electromagnetic waves, ultimately decreasing the antenna’s gain. The calculation of this area is illustrated in Figure 9.

In Figure 9, due to line symmetry, 1/6 of a module is selected for illustration. The area error resulting from the assembly gap is calculated as follows:

(4) $$S_{iJX}=3[(R+\frac{H}{2\cos\left(\frac{\theta }{2}\right)})(R\cos\left(\frac{\theta }{2}\right)+\frac{H}{2})-R^{2}\cos\left(\frac{\theta }{2}\right)]$$

where $S_{iJX}$ is the assembly gap error of the *i*-th module, H is the width of the docking mechanism, i.e., the dimension of the assembly gap.

### 2.3. Analysis of Errors

**Figure 10:**
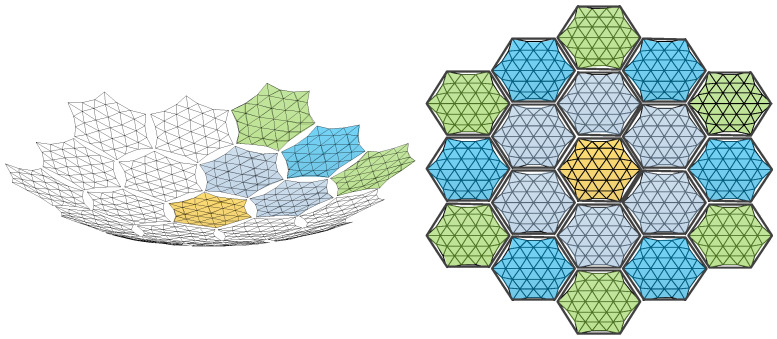
Diagram of an assembled antenna (only the mesh surface and the upper surface connection of supporting trusses are shown).

The primary error sources of the modular-assembled antenna include module surface errors, metal mesh area errors, and assembly gap errors, as shown in Figure 10. In the previous section, the definitions and calculations of each error were explained. In this section, we will discuss the impact of these errors on the overall performance of the modular-assembled antenna.

#### 2.3.1. Surface Errors of Assembled Antenna

The modular-assembled antenna is composed of multiple modules, meaning that the overall surface error is influenced by the surface error of each individual module. The surface error calculation for a single module is given in Equation (2). For the entire assembled antenna, its surface error depends not only on the errors of individual modules but also on the connections between them.

**Figure 11:**
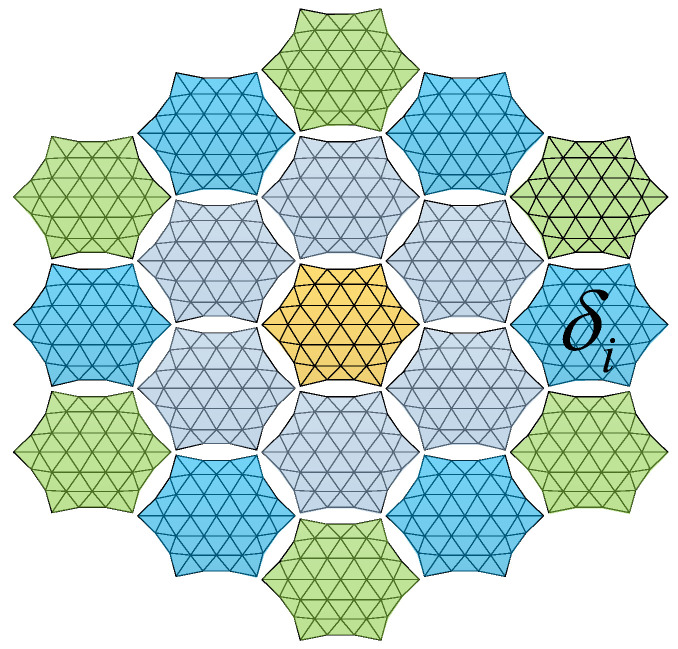
Model for calculating surface errors of assembled antenna.

To analyze the impact of single-module errors on the overall antenna surface, we assume an ideal assembly process with no area error in the cable network division. Thus, only the effect of mesh division within each module on the antenna surface is considered. Since the assembled antenna consists of various modules, the surface accuracy of modules at different locations can be independently designed. As illustrated in Figure 11, modules of the same color share identical surface accuracy.

The surface error of the assembled antenna shown in Figure 11 can be calculated as

(5) $$\delta_{RMS}=\sqrt{\frac{1}{N}\sum_{m=1}^{N}\delta_{m}^{2}}$$

where $\delta_{RMS}$ is the surface accuracy of the entire assembled antenna, $\delta_{m}$ is the surface accuracy of a single module unit, and N is the number of modules.

#### 2.3.2. Metal Mesh Area Error of Assembled Antenna

**Figure 12:**
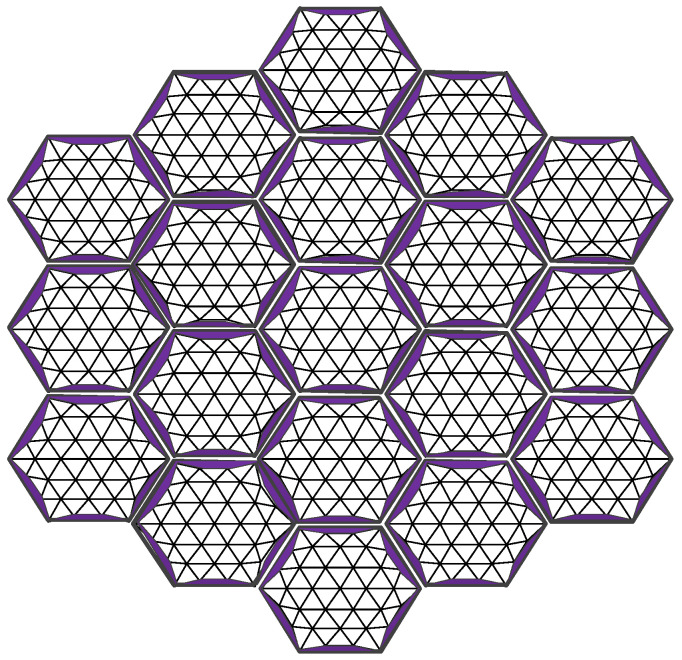
Metal mesh area error of assembled antenna.

The assembled antenna consists of a large number of modules, and as the number of modules increases, the metal mesh area error also accumulates. Therefore, for the assembled antenna, the metal mesh area error cannot be ignored. When analyzing the metal mesh area error, factors such as assembly gaps and mesh division are not considered. Instead, the focus is solely on the area deviation introduced by the metal mesh itself. The distribution of the metal mesh area error in the assembled antenna is illustrated in Figure 12.

In Figure 12, the metal mesh area error of a single module is defined by Equation (3). Based on this, the total metal mesh area error of the assembled antenna can be calculated as:

(6) $$S_{jsTX}=\sum_{i=1}^{N}S_{iJS}$$

where $S_{jsTX}$ is the area error of the metal mesh for the assembled antenna.

#### 2.3.3. Assembly Gap Error of Assembled Antenna

**Figure 13:**
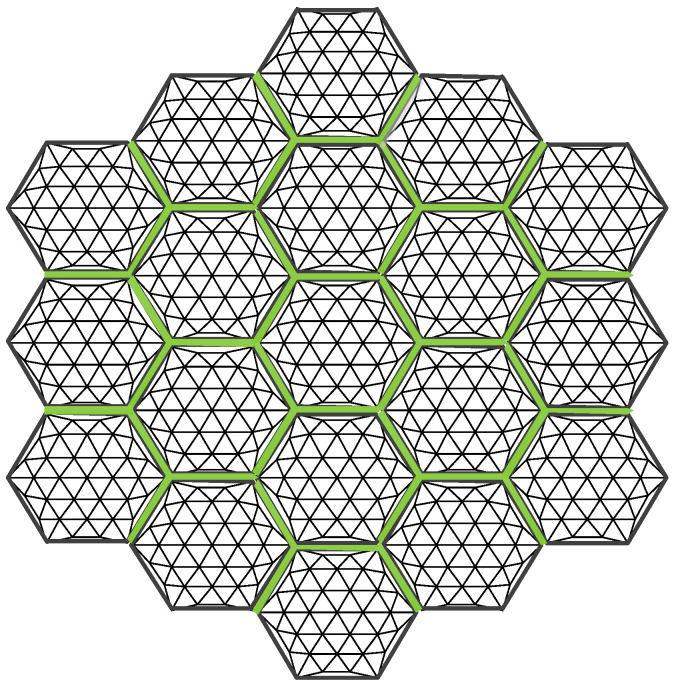
Overall antenna assembly gap area error.

During the module assembly process, the constraints imposed by the docking mechanism prevent a perfectly seamless assembly, resulting in assembly gaps between modules. These gaps appear on the antenna aperture surface as area loss, which degrades the overall performance of the assembled antenna. When analyzing the impact of assembly gap errors, the module reflector surface is assumed to be a standard parabolic surface, and only the effects of the assembly gaps are considered. The distribution of these gaps in the assembled antenna is illustrated in Figure 13.

From the assembly gap error of two modules in Equation (4), the assembly gap error of the entire assembled antenna can be described as

(7) $$S_{jsTX}=\sum_{i=1}^{N}S_{iJX}$$

where $S_{jsTX}$ is the assembly gap area error for the antenna.

### 2.4. Analysis of the Impact on Radiation Characteristics

Different error sources in a modular-assembled antenna create discrepancies between the actual antenna and the ideal reflector, leading to the degradation of its electrical performance. To evaluate the impact of these errors, an analysis and simulation of the antenna’s electrical performance is conducted, focusing on the three primary error sources: surface errors, metal mesh area errors, and assembly gaps. Based on electromagnetic field theory and engineering experience, the relationship between antenna gain and reflector surface accuracy can be estimated using the following formula [34]:

(8) $$∆G=10lg(e^{-{\left(\frac{4\pi \delta }{\lambda }\right)}^{2}})$$

The relationship between antenna gain and the aperture area of the reflector is given by:

(9) $$G_{0}=10lg(\frac{4\pi }{\lambda^{2}}\eta S)$$

where ∆G is gain loss due to reflector surface error, $G_{0}$ is ideal reflecting surface gain, δ is the accuracy of the reflector surface, λ is the wavelength, η is antenna efficiency, S is the reflector area.

## 3. Simulation Analysis

**Figure 14:**
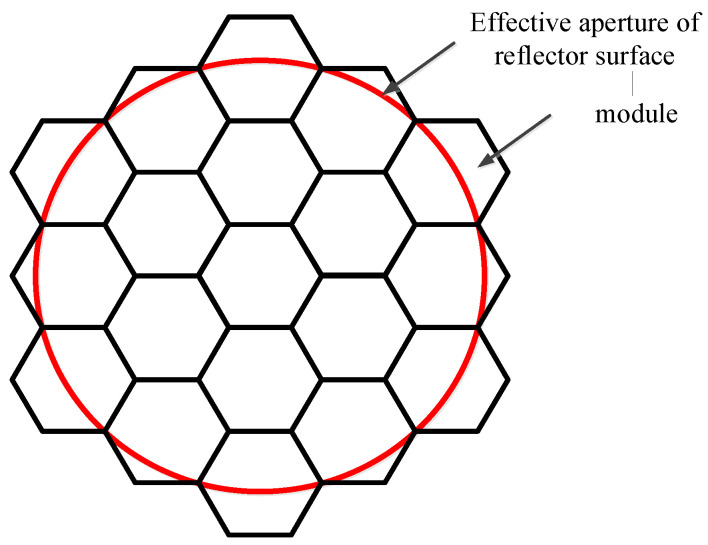
The effective aperture of the modular and the reflector surface.

From the above equations, it is evident that various factors influence the reflector gain, and their effects can be quantified using established calculation methods. However, due to the complexity of these interactions, professional finite element analysis (FEA) software (TICRA Tools 19.0) is required for accurate simulation and analysis. To investigate the impact of different error sources on the electrical performance of a modular-assembled antenna, a case study is conducted through simulation. The parameters of the reflector used in the study are presented in Table 1, while the reflector module configuration and effective aperture distribution are illustrated in Figure 14.

**Figure 15:**
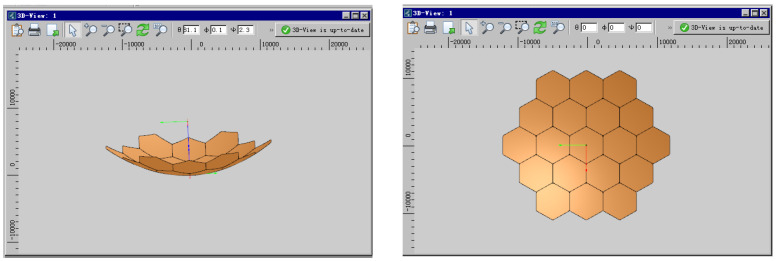
Electromagnetic simulation case of the modular-assembled antenna.

**Figure 16:**
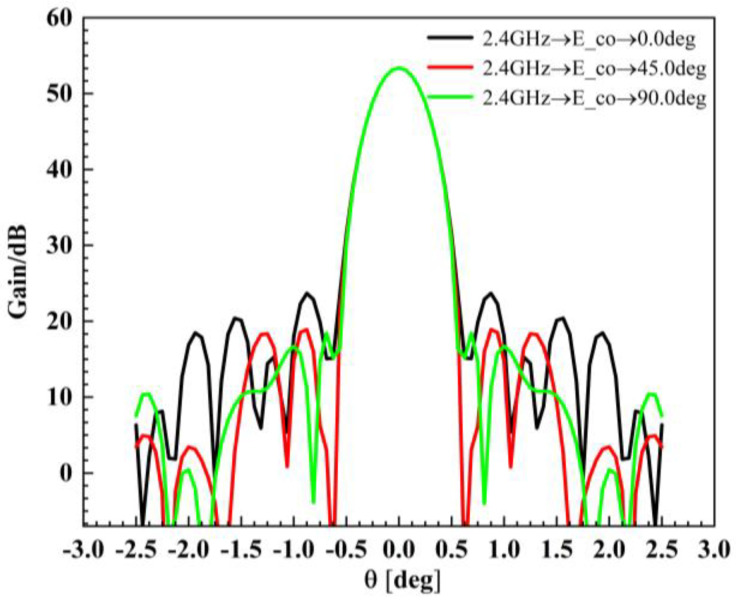
Antenna simulation results (standard module).

To investigate the impact of various error sources on the performance of the assembled antenna, a Gaussian feed is selected. During the analysis, both the antenna feed and focal length remain unchanged to ensure consistency. As a baseline for error evaluation, an initial simulation is conducted using an ideal module configuration, where all 19 modules in Figure 14 are assumed to be perfect parabolic surfaces. This serves as a reference for comparing the effects of surface errors, metal mesh area errors, and assembly gaps. Figure 15 illustrates the electromagnetic simulation model used in the analysis, while the corresponding simulation results are presented in Figure 16.

The simulation results of the standard parabolic surface are shown in Figure 16, which consists of 19 modules. Each module is generated by projecting a horizontal regular hexagon onto the parabolic surface, as shown in Figure 15.

### 3.1. Analysis of Surface Error and Radiation Characteristics

**Figure 17:**
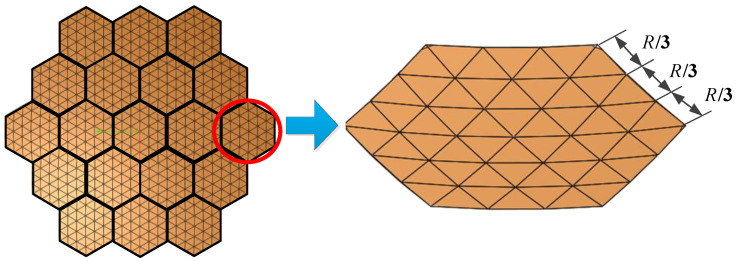
Module mesh division in the electromagnetic model.

**Figure 18:**
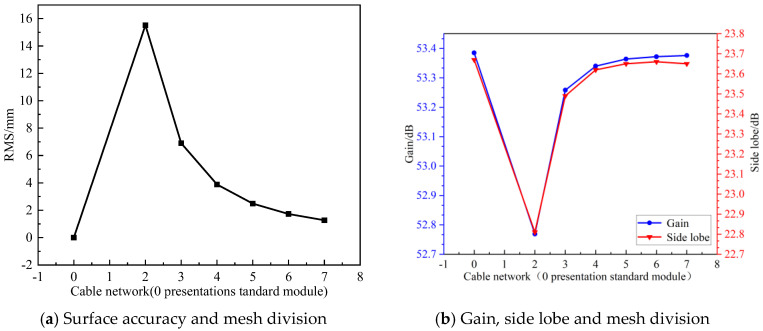
Relationship between gain, side lobe and surface accuracy.

The surface error of the modular-assembled antenna is directly influenced by the surface accuracy of each module. Different module units can employ various mesh division strategies in their cable network design. However, to maintain consistency across all modules, a uniform division method is applied in this study. As an example, Figure 17 illustrates a case where each module is divided into three sections. Once the mesh division is established, the overall surface accuracy of the assembled antenna is calculated. Subsequently, its electrical performance is simulated to evaluate the impact of surface errors. In this analysis, the standard surface is defined as a configuration in which each module is a perfect parabolic surface with no errors. Thus, the corresponding surface accuracy for this ideal case is 100% accurate. The simulation results for the standard surface serve as theoretical reference values. All other simulation results, which incorporate different surface error conditions, are compared against these reference values to assess performance degradation. The comparative analysis of these results is presented in Figure 18.

As shown in Figure 18, the surface accuracy of individual modules has a significant impact on the antenna’s gain compared to the standard parabolic surface. A higher surface accuracy, achieved through increased mesh division, results in a larger gain. However, once the surface accuracy reaches a certain threshold (λ/30 is selected on engineering standards), the gain increase becomes negligible, approaching the theoretical gain of a perfect standard module.

Beyond this threshold, further improving surface accuracy does not provide substantial performance benefits but instead leads to higher manufacturing costs, making it less favorable for practical engineering applications. Additionally, while sidelobe levels initially increase with higher surface accuracy, they eventually stabilize and align with those of the standard parabolic surface.

In the engineering application of modular-assembled antennas, it is crucial to strike a balance between gain and sidelobe levels during surface design. This ensures that the antenna meets the required performance specifications while also minimizing the complexity of module design and manufacturing costs.

### 3.2. Analysis of Metal Mesh Area Error and Radiation Characterization

In engineering applications, mesh antennas rely on a metal mesh attached to a tension cable-net to reflect electromagnetic waves. The projection area of the metal mesh on the antenna aperture plays a critical role in determining the radiation characteristics of the antenna. For modular-assembled antennas, each module is designed as a hexagonal prism capable of folding and deploying. These modules connect to the tension cable-net only at the six vertices of the hexagon. To ensure structural stability, the tension cable-net is designed with a specific sag-to-span ratio. However, this design constraint prevents the metal mesh from fully covering the aperture surface of each module, leading to electromagnetic leakage. When a large number of modules are assembled, these area errors accumulate, significantly affecting the overall electrical performance of the antenna.

**Figure 19:**
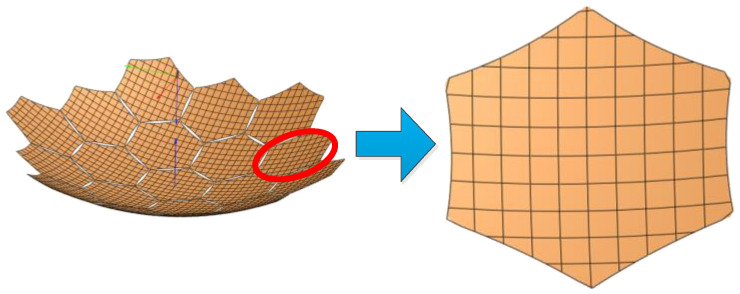
Area error of metal mesh in electromagnetic model (sag-to-span ratio).

**Figure 20:**
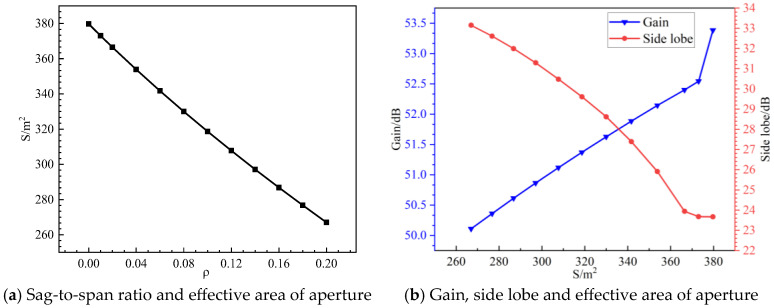
The relationship between gain, side lobe level, aperture surface effective area, and sag-to-span ratio.

Figure 19 illustrates the metal mesh area error. To analyze this error, each module is assumed to be a perfect parabolic surface with no surface errors or assembly gap errors. The metal mesh area error is primarily influenced by the sag-to-span ratio of the mesh surface. To assess its impact, simulations of the radiation characteristics of modular-assembled antennas are conducted for different sag-to-span ratios. A sag-to-span ratio of 0 represents an ideal case where each module perfectly conforms to a parabolic shape. The simulation results for this scenario serve as theoretical reference values. The remaining simulation results, corresponding to different sag-to-span ratios, are then compared against these reference values, as shown in Figure 20.

As shown in Figure 20, the sag-to-span ratio of the module has a significant impact on the antenna’s performance compared to the standard parabolic surface.

- When the metal mesh area is no less than 353.84 m^2^ (0<ρ<0.04), the side lobes increase slowly, the effective aperture area changes slightly, and the gain decreases by less than 1 dB. This indicates that within this range, the sag-to-span ratio has a relatively minor impact on antenna performance.
- However, as the sag-to-span ratio increases, the effective aperture area decreases rapidly, leading to a notable drop in antenna gain.
- When the sag-to-span ratio reaches 0.2, the antenna gain decreases by 3.275 dB, resulting in an over 50% loss of radiated energy. At the same time, the side lobes rise sharply, significantly affecting the antenna’s directivity and signal quality.

These results highlight the critical influence of metal mesh area errors on antenna performance. As the aperture surface area of the metal mesh decreases, the radiation characteristics deteriorate rapidly. Therefore, in the engineering design of modular-assembled antennas, careful selection of the sag-to-span ratio is essential, as it directly impacts the effective metal mesh area and, consequently, the antenna’s overall performance.

### 3.3. Analysis of Assembly Gap Error and Radiation Characterization

The assembly gap is a critical factor affecting the performance of modular-assembled antennas. The docking mechanism, essential for module assembly, introduces unavoidable gaps between modules due to its structural dimensions. These gaps lead to a reduction in the effective aperture area of the antenna, ultimately degrading its performance.

**Figure 21:**
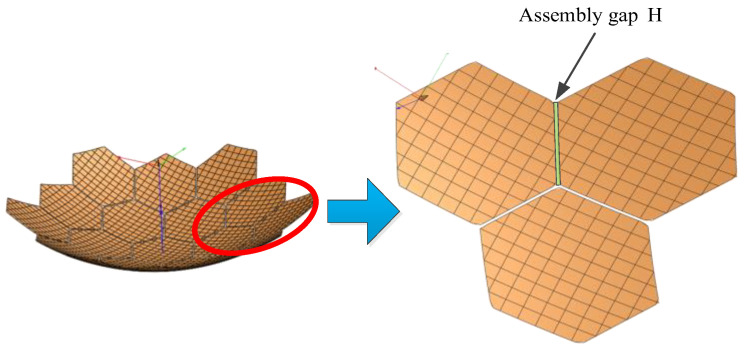
Assembly gap error in the electromagnetic models.

Figure 21 illustrates the assembly gap error in a modular-assembled antenna. To isolate the impact of assembly gaps, the analysis does not consider metal mesh area errors and surface accuracy errors. Instead, each module is assumed to be a perfect parabolic surface, and only the performance degradation caused by the assembly gaps is examined.

**Figure 22:**
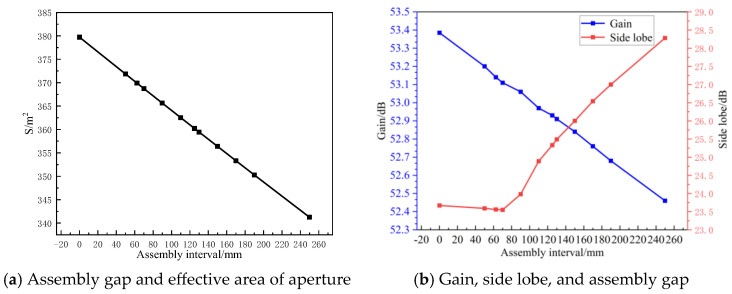
Relation between gain, side lobe electrical level, aperture area and assembly gap.

Simulations are conducted for different assembly gap sizes to evaluate their effect on the electrical performance of the antenna. An assembly gap of 0 represents an ideal case where all modules are seamlessly connected, serving as the theoretical reference value. The simulation results corresponding to different assembly gap sizes are then compared against this reference, with the findings presented in Figure 22.

As shown in Figure 22, the assembly gap significantly affects both the gain and side lobes of the antenna compared to the standard parabolic surface.

- As the assembly gap increases, the effective aperture area decreases, leading to a reduction in antenna gain. When the assembly gap is approximately half a wavelength (λ/2), the gain reduction is minimal, and the side lobes reach their lowest value, indicating an optimal balance between gain and interference control.
- However, as the assembly gap continues to increase, the side lobes rise rapidly, and the difference between gain and side lobes decreases, leading to worsened directivity and increased interference sensitivity.
- When the assembly gap is 2λ, the antenna gain decreases by nearly 1 dB, while side lobes increase by 5 dB, confirming a decline in the antenna’s directivity and anti-interference capability.

Since larger assembly gaps degrade antenna performance due to the loss of effective aperture area, maintaining an optimal assembly gap near λ/2 ensures better electrical performance. Therefore, during the design of modular-assembled antennas, the docking mechanism dimensions should be carefully controlled to be as close as possible to half the wavelength of the operating frequency band.

## 4. Conclusions

In this paper, the error definitions of modular units in a modular-assembled antenna are proposed, and the mathematical formulation for each error type is derived. Subsequently, the relationship between modular-assembled antenna errors and individual module errors is analyzed, leading to the derivation of the overall error for the assembled antenna. Finally, the impact of the three major error sources on the electrical performance of the modular-assembled antenna is evaluated, resulting in the following conclusions:

### 4.1. Surface Accuracy Impact

The surface accuracy of the modular-assembled antenna significantly influences both gain and side lobe levels. When the surface accuracy reaches a certain threshold, the gain and side lobes become comparable to those of a standard parabolic surface. Beyond this point, further improvements in surface accuracy provide negligible performance benefits but significantly increase design and manufacturing complexity in engineering applications.

### 4.2. Metal Mesh Area Error Impact

The metal mesh area error plays a crucial role in determining the electrical performance of the antenna, and it is primarily governed by the sag-to-span ratio. When the sag-to-span ratio remains within the range 0<ρ<0.04, the antenna gain and side lobe levels remain practically unaffected. However, as the sag-to-span ratio increases, the effective aperture area decreases rapidly, leading to a sharp degradation in electrical performance. When the sag-to-span ratio reaches 0.2, the radiated energy decreases by more than 50%, severely impacting antenna efficiency. Therefore, careful selection of the sag-to-span ratio is essential in the design of modular-assembled antennas.

### 4.3. Assembly Gap Impact

The assembly gap affects both antenna gain and side lobes. As the assembly gap increases, the antenna gain decreases, and the side lobe levels rise, deteriorating directivity and signal quality. When the assembly gap approaches the λ/2 of the S-band, the side lobe levels are minimized, and the antenna achieves optimal electrical performance. Therefore, during the design phase, the dimensions of the docking mechanism should be carefully controlled, ensuring that its physical size is as close as possible to the target wavelength for optimal performance.

## Figures and Tables

**Table 1:** Parameters of reflector surface.

Name	Focal Length	Effective Aperture	Number of Modules	Frequency Band
Parameter	8 m	20 m	19	2.4 GHz

## Data Availability

Data are contained within the article.
